# Optimal imaging time points considering accuracy and precision of Patlak linearization for ^89^Zr-immuno-PET: a simulation study

**DOI:** 10.1186/s13550-022-00927-6

**Published:** 2022-09-05

**Authors:** Jessica E. Wijngaarden, Marc C. Huisman, Johanna E. E. Pouw, C. Willemien Menke-van der Houven van Oordt, Yvonne W. S. Jauw, Ronald Boellaard

**Affiliations:** 1 Department of Radiology and Nuclear Medicine, Amsterdam UMC location Vrije Universiteit Amsterdam, Boelelaan 1117, Amsterdam, The Netherlands; 2 Department of Medical Oncology, Amsterdam UMC location Vrije Universiteit Amsterdam, Boelelaan 1117, Amsterdam, The Netherlands; 3 Department of Hematology, Amsterdam UMC location Vrije Universiteit Amsterdam, Boelelaan 1117, Amsterdam, The Netherlands; 4 Cancer Center Amsterdam, Imaging and Biomarkers, Amsterdam, The Netherlands

**Keywords:** ^89^Zr-immuno-PET, Patlak linearization, Monoclonal antibody, Molecular imaging

## Abstract

**Purpose:**

Zirconium-89-immuno-positron emission tomography (^89^Zr-immuno-PET) has enabled visualization of zirconium-89 labelled monoclonal antibody (^89^Zr-mAb) uptake in organs and tumors in vivo. Patlak linearization of ^89^Zr-immuno-PET quantification data allows for separation of reversible and irreversible uptake, by combining multiple blood samples and PET images at different days. As one can obtain only a limited number of blood samples and scans per patient, choosing the optimal time points is important. Tissue activity concentration curves were simulated to evaluate the effect of imaging time points on Patlak results, considering different time points, input functions, noise levels and levels of reversible and irreversible uptake.

**Methods:**

Based on ^89^Zr-mAb input functions and reference values for reversible (*V*_*T*_) and irreversible (*K*_*i*_) uptake from literature, multiple tissue activity curves were simulated. Three different ^89^Zr-mAb input functions, five time points between 24 and 192 h p.i., noise levels of 5, 10 and 15%, and three reference *K*_*i*_ and *V*_*T*_ values were considered. Simulated *K*_*i*_ and *V*_*T*_ were calculated (Patlak linearization) for a thousand repetitions. Accuracy and precision of Patlak linearization were evaluated by comparing simulated *K*_*i*_ and *V*_*T*_ with reference values.

**Results:**

Simulations showed that *K*_*i*_ is always underestimated. Inclusion of time point 24 h p.i. reduced bias and variability in *V*_*T*_, and slightly reduced bias and variability in *K*_*i*_, as compared to combinations of three later time points. After inclusion of 24 h p.i., minimal differences were found in bias and variability between different combinations of later imaging time points, despite different input functions, noise levels and reference values.

**Conclusion:**

Inclusion of a blood sample and PET scan at 24 h p.i. improves accuracy and precision of Patlak results for ^89^Zr-immuno-PET; the exact timing of the two later time points is not critical.

**Supplementary Information:**

The online version contains supplementary material available at 10.1186/s13550-022-00927-6.

## Introduction

Therapeutic monoclonal antibodies (mAbs) are used in cancer treatment both in targeted therapy and in immunotherapy [1]. mAbs directly elicit their effect on their target or indirectly through mediation by the immune system. The effectiveness of this therapy is, however, patient specific and the therapy can cause serious side effects. Gaining more insight into the mechanisms of mAbs by tracking them inside the body may improve cancer treatment with mAbs.

Zirconium-89-immuno-positron emission tomography (^89^Zr-immuno-PET) allows visualization and quantification of the uptake of zirconium-89 labelled mAbs (^89^Zr-mAbs) in tumors and organs in vivo. The relatively long half-life of ^89^Zr is sufficient for imaging mAbs during the time they need to reach tissues [2]. Quantification of ^89^Zr-mAb uptake is commonly done using the standardized uptake value (SUV). SUV is defined as the activity concentration in a volume of interest, divided by the injected activity per unit of body weight [3]. Since SUV is a single value obtained from a single PET scan, SUV is not able to distinguish between non-specific ^89^Zr-mAb uptake in the blood or interstitial space volume fraction of the tissue, and specific uptake due to target engagement, unless either specific or non-specific uptake can be assumed to be negligible. In general, both non-specific and specific uptake contribute to the total uptake signal. Additionally, SUV considers only the injected activity and not the ^89^Zr-mAb plasma clearance over time [4].

An approach that does consider plasma activity concentrations for analyzing PET images is the use of compartment models [5]. Using a two-tissue compartment model assuming irreversible uptake of tracer, Patlak linearization can be applied [6]. A two-tissue irreversible compartment model is applicable to ^89^Zr-mAb uptake, because ^89^Zr residualizes in the tissue after mAb catabolism or target engagement [2]. The uptake of ^89^Zr-mAbs in tissue is quantified relative to the concentration of ^89^Zr-mAbs in blood plasma over time and therefore requires multiple blood samples and PET images. Since ^89^Zr-mAbs circulate in the body for several days [7], capturing the pharmacokinetics of ^89^Zr-mAbs requires multiple sampling days. However, minimizing the number of scans and samples is important in terms of patient safety and comfort. Selecting the optimal time points for blood sampling and PET imaging of ^89^Zr-mAbs is therefore crucial.

Patlak linearization provides several advantages over SUV. From Patlak linearization, reversible and irreversible ^89^Zr-mAb uptake can be quantified per volume of interest. Additionally, Patlak can potentially also distinguish between non-specific and specific ^89^Zr-mAb uptake, by comparing Patlak results to baseline Patlak values for tissues without target expression [8]. Moreover, Patlak linearization uses the measured plasma kinetics and thus takes variations in plasma clearance between subjects or at various mass doses into account. Yet, like SUV, Patlak linearization assumes that receptor availability or occupancy remains constant during the course of the PET studies and does not consider redistribution of cells or targets, as will be discussed later.

Previous research has applied Patlak linearization for quantifying ^89^Zr-mAbs uptake in patients [8, 9]. In these studies, PET scans were obtained two to four times between 2 and 192 h p.i. Blood was sampled up to five times on the day of injection and with every PET scan [8, 9]. This resulted in a maximum of three time points for Patlak linearization. The unavoidable sparse data sampling introduces uncertainties in the data which may affect Patlak results. Evaluating the magnitude of the effects of sparse data sampling will provide more information on the accuracy and precision of Patlak results.

In this study, the effect of imaging time points on the accuracy and precision of Patlak results was evaluated by means of simulations, including the following variables: different input functions (IFs), different noise levels for tissue activity curves (TACs) and tissues with different levels of reversible and irreversible uptake.

## Methods

To study the effects of different time points on Patlak results, TACs were simulated using Patlak linearization, three time points were included, noise was added and Patlak values were calculated. These steps were repeated as a function of different variables.

### Patlak linearization

Patlak linearization can be used to estimate the irreversible and reversible uptake of ^89^Zr-mAb in tissue based on graphical analysis of multiple-time tissue uptake data [6]. The analysis is based on a compartment model consisting of a reversible and an irreversible tissue compartment. The reversible tissue compartment represents ^89^Zr-mAb in the plasma and interstitial space of the tissue or reversible target binding, and reaches an equilibrium state after some time. The irreversible tissue compartment represents irreversible binding of ^89^Zr-mAb (e.g., non-specific catabolism or irreversible target binding). After equilibrium is reached, the activity concentration in tissue (AC_*t*_) is the sum of both parts. The reversible part is then proportional to the activity concentration in plasma (AC_*p*_) and the irreversible part is proportional to the area under the curve (AUC) of the AC_*p*_ (AUC_*p*_), which is the integral of AC_*p*_ (Eq. 1). Dividing both sides of Eq. 1 by AC_*p*_ results in a linear relation known as the Patlak equation (Eq. 2) [6, 9]. The slope of this equation is *K*_*i*_, which represents the nett rate of irreversible uptake [h ^−1^]. *K*_*i*_ is a measure for the catabolic rate of tissue without target expression and a measure for both catabolic rate and target engagement of tissue with target expression [8]. The offset is the *V*_*T*_, the ratio between tissue and plasma concentration at equilibrium, which is related to the reversible part. (Eq. 2).

1 $${\text{AC}}_{t} = K_{i} \cdot {\text{AUC}}p + V_{T} \cdot {\text{AC}}_{p}$$

2 $$\frac{{{\text{AC}}_{t} }}{{{\text{AC}}_{p} }} = K_{i} \cdot \frac{{\mathop \smallint \nolimits_{0}^{t} {\text{AC}}_{P} \left( x \right){\text{d}}x}}{{{\text{AC}}_{P} }} + V_{T}$$

**Fig.1:**
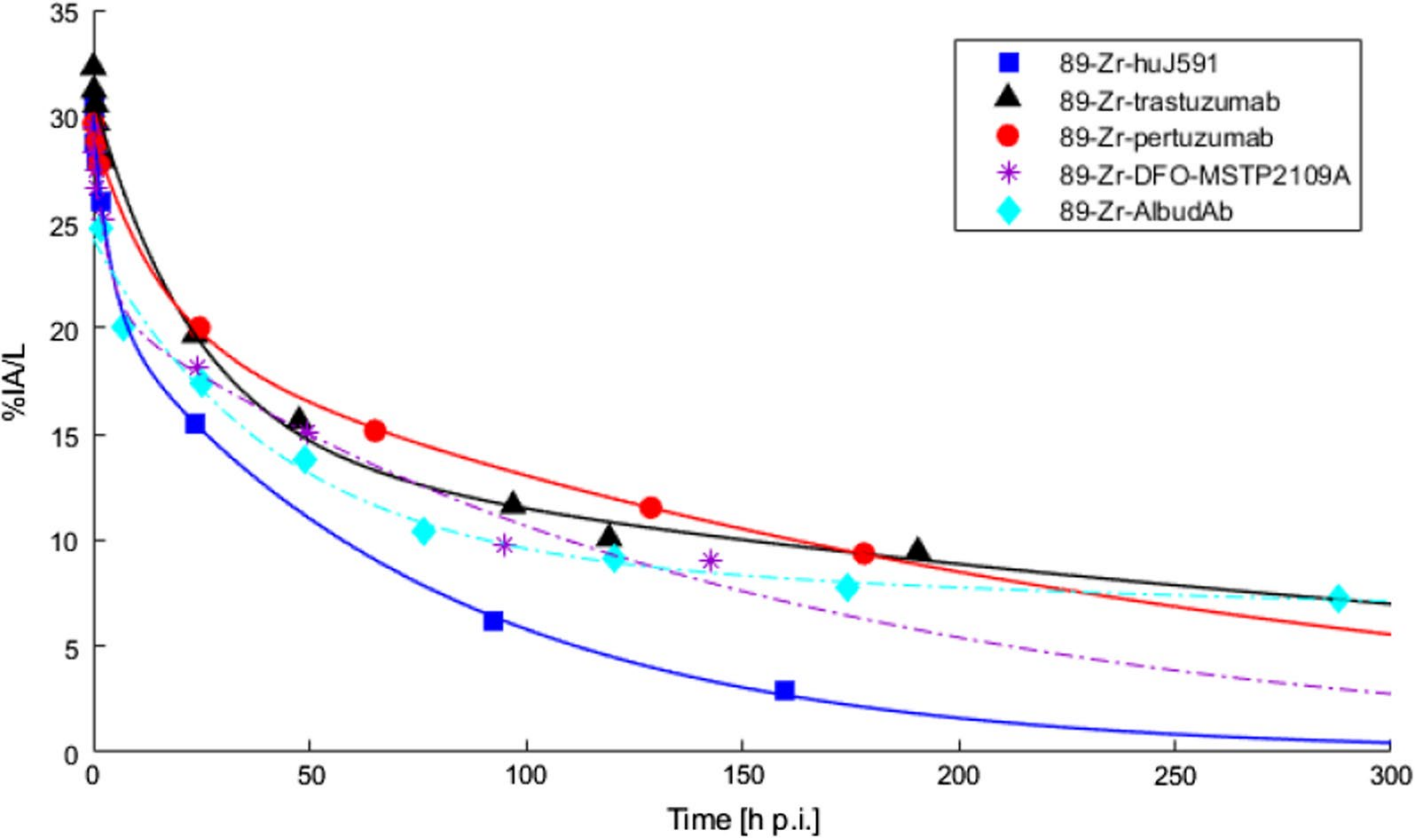
Plasma or serum activity concentrations in percentage injected activity per liter as a function of time in hours post-injection for ^89^Zr-huJ591 (191.3 ± 9 MBq, 25 mg) [10], ^89^Zr-trastuzumab (185 MBq, 50 mg) [11], ^89^Zr-pertuzumab (74 MBq, 20 or 50 mg) [12], ^89^Zr-DFO-MSTP2109A (184 MBq, 10 mg) [13] and ^89^Zr-AlbudAb (14 MBq, 1 mg) [14]. The bold lines represent the input functions used for the simulations, and the dashed lines represent the input functions not included in the simulations. %IA/L = percentage injected activity per liter, h p.i. = hours post-injection

Multiple population IFs were obtained from literature as input for the AC_*p*_. A literature search for papers containing plasma/serum sampling data of ^89^Zr-mAb concentration in humans resulted in five papers as listed in Table 1. From these papers, the concentration ^89^Zr labelled mAbs in plasma/serum over time was obtained using PlotDigitizer (version 2.6.8, http://plotdigitizer.sourceforge.net/). The purpose of using IFs from literature was to use IFs that could be obtained in practice. Therefore, instead of using the raw data points, a bi-exponent (Eq. 3) was fitted through the data, see Fig. 1. The concentrations of the three IFs ^89^Zr-trastuzumab, ^89^Zr-pertuzumab and ^89^Zr-huJ591 were chosen as input for AC_*p*_ in the simulations, as they presented three different clearance rates.

**Table 1:** Five papers provided ^89^Zr-mAb plasma/serum activity concentration data

	^89^Zr-mAb	Subject group	Sampling	References
1	^89^Zr-huJ591	Metastatic prostate cancer	Serum	Pandit-Taskar et al. [10]
2	^89^Zr-trastuzumab	Esophagogastric cancer	Serum	O’Donoghue et al. [11]
3	^89^Zr-pertuzumab	Breast cancer	Serum	Ulaner et al. [12]
4	^89^Zr-DFO-MSTP2109A	Prostate cancer	Plasma	O’Donoghue et al. [13]
5	^89^Zr-AlbudAb	Healthy volunteers	Plasma	Thorneloe et al. [14]

The Patlak equation is used to simulate AC_*t*_ as function of AC_*p*_, *K*_*i*_ and *V*_*T*_, i.e., to generate TAC_*s*_. The given *K*_*i*_ and *V*_*T*_ for generating the TAC are called ‘reference *K*_*i*_ (rK_i_)’ and ‘reference *V*_*T*_ (*rV*_*T*_)’. The mathematical derivation for the TAC is as follows. AC_*p*_ is described by a bi-exponential function (Eq. 3). AUC_*p*_ can be obtained by integration of Eq. 3 between moment of injection and moment of PET scan, resulting in Eq. 4. Substitution of Eqs. 3 and 4 into Eq. 1 gives the equation for the TAC (AC_*t*_) as a function of *rK*_*i*_, *rV*_*T*_ and coefficients of the bi-exponential equation of the IF (Eq. 5):

3 $${\text{AC}}_{p} = A \cdot {\text{e}}^{ax} + B \cdot {\text{e}}^{bx}$$

4 $${\text{AUC}}p = \mathop \smallint \limits_{0}^{t} \left( {A \cdot {\text{e}}^{ax} + B \cdot {\text{e}}^{bx} } \right){\text{d}}x = \frac{{A \cdot \left( {{\text{e}}^{ax} - 1} \right)}}{a} + \frac{{B \cdot \left( {{\text{e}}^{bx} - 1} \right)}}{b}$$

5 $${\text{AC}}_{t} = rK_{i} \cdot \left( {\frac{{A \cdot \left( {{\text{e}}^{ax} - 1} \right)}}{a} + \frac{{B \cdot \left( {{\text{e}}^{bx} - 1} \right)}}{b}} \right) + rV_{t} \cdot \left( {A\cdot{\text{e}}^{ax} + B \cdot {\text{e}}^{bx} } \right)$$

### Sparse sampling and noise

**Fig. 2:**
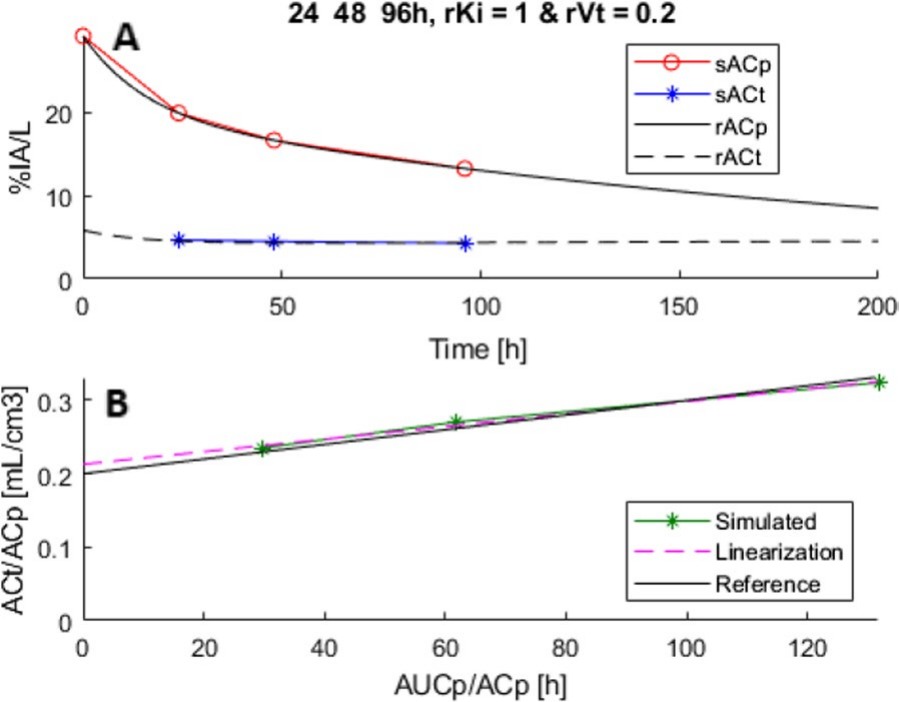
Patlak linearization for ^89^Zr-pertuzumab input function and time activity curves with 5% noise, *rK*_*i*_ = 1·10^−3^ [h^−1^], *rV*_*T*_ = 0.2 and time points 24, 48 and 96 h post-injection. **A**: Activity concentrations in plasma (red) and tissue (blue), full curve based on reference values (rAC_*p*_ and rAC_*t*_) and calculated based on simulations (sAC_*p*_ and sAC_*t*_) in percentage injected activity per liter as a function of time p.i. **B**: Patlak plot; activity concentration in tissue (AC_*t*_) divided by activity concentration in plasma (AC_*p*_) as a function of area under the plasma curve (AUC_*p*_) divided by activity concentration in plasma (AC_*p*_). Based on reference values (black), simulated values (green) and linear regression of the simulated values (pink). %IA/L = percentage injected activity per liter

For a given IF, *rK*_*i*_ and *rV*_*T*_, values for AC_*p*_ and AC_*t*_ were determined with the equations above on three given time points, mimicking the sparse sampling in practice. AUC_*p*_ was determined, but now by numerical integration of the IF, considering only four time points of AC_*p*_ (see red line first panel Fig. 2). Additionally, noise was added to values for AC_*t*_ at the given three time points. Standard deviations (SDs) of AC_*t*_ were approximated based on counting statistics, which behaves as a Poisson distribution with SD≈√N and N is number of counts [15]. The SD at any given time point was approximated with Eq. 6, where the SD at *t* = 0 is predefined. Assuming equal scanning durations within a study, the ratio N(0):N(t) is assumed to be equal to the ratio between non-decay corrected activity concentrations ncAC_*t*_(0):ncAC_*t*_(t) (Eq. 7). To incorporate variability in the standard deviation, noise was added using the MATLAB function randn [16]. Subsequently, the percentage SD was calculated and applied on the decay corrected AC_*t*_ for adding noise to AC_*t*_ (Eq. 8).

6 $${\text{SD}}\left( t \right) = \frac{{{\text{SD}}\left( 0 \right)}}{{\sqrt {N\left( 0 \right)/N\left( t \right)} }}$$

7 $${\text{SD}}\left( t \right) = \frac{{{\text{SD}}\left( 0 \right)}}{{\sqrt {{\text{ncAC}}_{t} \left( 0 \right)/{\text{ncAC}}_{t} \left( t \right)} }}$$

8 $$AC_{t,noise} \left( t \right) = AC_{t} \left( t \right) + AC_{t} \left( t \right)*\% SD\left( t \right)*randn$$

Variability in AC_*p*_ as a result of counting statistics ranged from SD = 0.2–0.4%, based on previously in house counted blood samples. The noise in AC_*p*_ was assumed to be negligible compared to the noise in the TAC and was not included in the simulations.

### Patlak analysis of simulated TACs

Subsequently, Patlak linearization (Eq. 2) was applied on the generated AC_*p*_, AUC_*p*_ and AC_*t*_ with noise on the given time points, from which the slope (*K*_*i*_) and offset (*V*_*T*_) could be determined, see Fig. 2. Simulations were repeated 1000 times to incorporate the effect of noise. The mean and standard error (SE) of the simulated *K*_*i*_ and *V*_*T*_ were obtained to compare with *rK*_*i*_ and *rV*_*T*_ for evaluating bias and variability.

### Performance of Patlak analysis

Accuracy and precision of Patlak results were evaluated as a function of the following variables: time points of evaluation, *rK*_*i*_ and *rV*_*T*_, and noise level of AC_*t*_. Each simulation included a time point at 0 h p.i. for AC_*p*_. Additionally, three of the following time points in hours post-injection were considered: 24, 48, 96, 144 and 192, which resulted in 10 time point combinations. The chosen values for *rK*_*i*_ were 1, 5 and 20 ∙10^–3^ h^−1^, representing real values of *K*_*i*_ for tissue without target expression [8], and two levels of target expression, respectively. The chosen *rV*_*T*_ were 0.1, 0.2 and 0.5. These values were comparable to baseline values for *V*_*T*_ as found by Jauw et al. [8], which agreed with predicted values for *V*_*T*_ as sum of antibody biodistribution coefficient [17] and the plasma volume fraction. The noise levels of the TAC at time 0 were varied from 5%, 10% to 15%, equal to noise levels for the TAC previously used in a Patlak simulation study [18]. Simulations were performed in MATLAB (v9.3.0.713579) [16] using in-house written code (see Additional file 1).

## Results

**Fig. 3:**
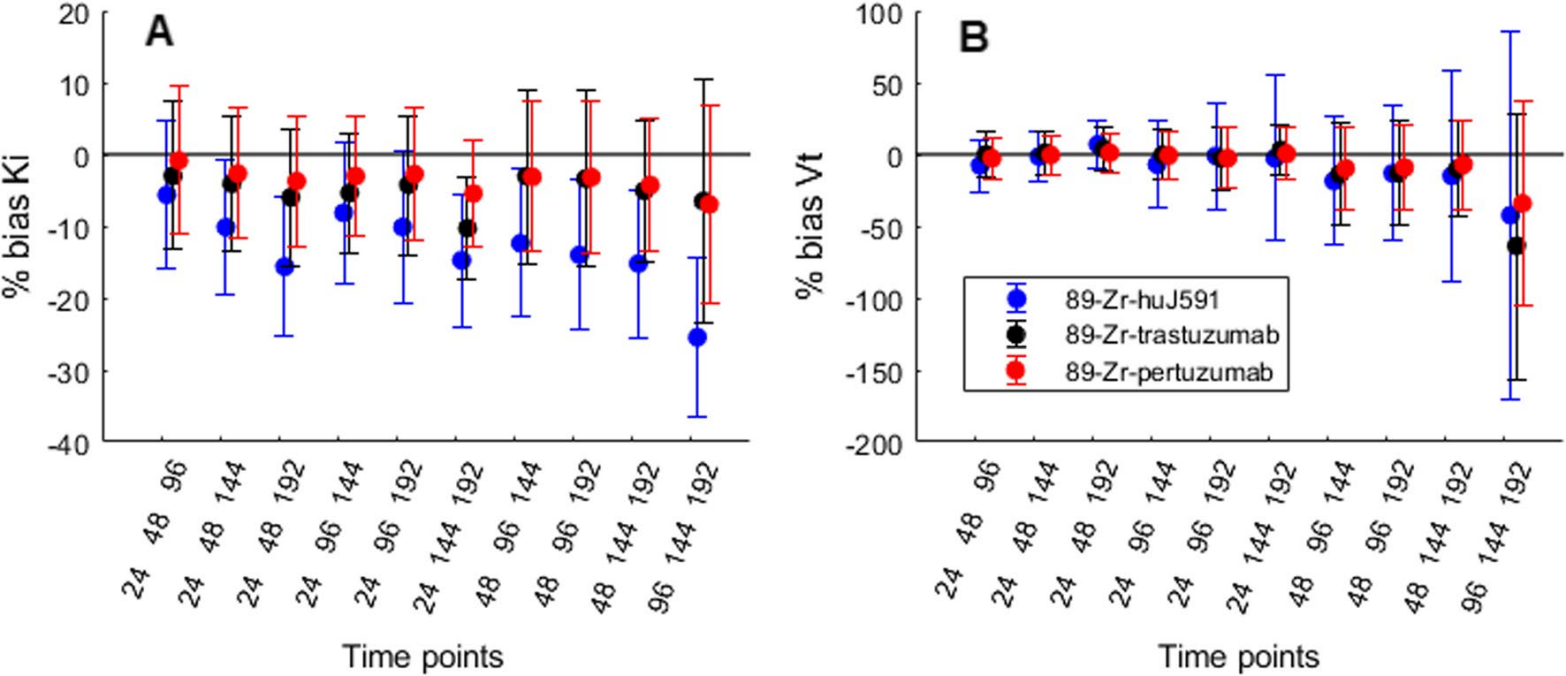
Percentage bias and variability of *K*_*i*_ (**A**) and *V*_*T*_ (**B**) per time point combination, for ^89^Zr-huJ591, ^89^Zr-trastuzumab and ^89^Zr-pertuzumab input functions and time activity curves with 5% noise, *rK*_*i*_ = 5·10^−3^ [h^−1^] and *rV*_*T*_ = 0.2. Combinations including 24 h post-injection showed smaller bias and variability than combinations without 24 h p.i. *rK*_*i*_ = reference *K*_*i*_, *rV*_*T*_ = reference *V*_*T*_

**Fig. 4:**
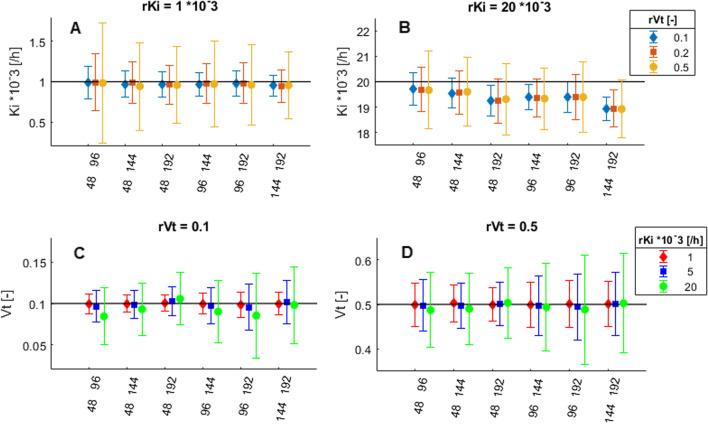
Absolute *K*_*i*_ (**A** and **B**) and *V*_*T*_ (**C** and **D**) values per time point combination for *rK*_*i*_ = 1 and 20·10^–3^ [h^−1^], and for *rV*_*T*_ = 0.1 and 0.5, for ^89^Zr-pertuzumab input function and time activity curves with 5% noise. All time point combinations on the x-axis also included 24 h post-injection. *rK*_*i*_ = reference *K*_*i*_, *rV*_*T*_ = reference *V*_*T*_

**Fig. 5:**
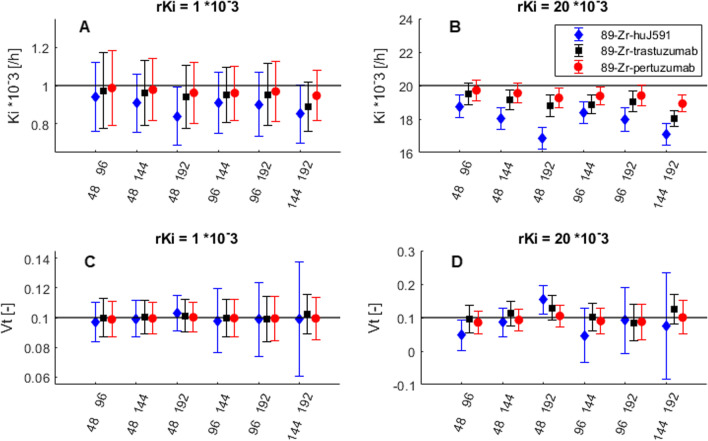
Absolute *K*_*i*_ (**A** and **B**) and *V*_*T*_ (**C** and **D**) values per time point combination for ^89^Zr-huJ591, ^89^Zr-trastuzumab and ^89^Zr-pertuzumab input functions, with *rK*_*i*_ = 1 and 20·10^−3^ [h^−1^], *rV*_*T*_ = 0.1 and time activity curves with 5% noise. All time point combinations on the x-axis also included 24 h post-injection. *rK*_*i*_ = reference *K*_*i*_, *rV*_*T*_ = reference *V*_*T*_

Simulations showed that bias in *K*_*i*_ was negative in all situations, see Figs. 3, 4 and 5 and Table 2. Inclusion of a time point at 24 h p.i. improved accuracy and precision of Patlak results in almost all simulations. Simulations with ^89^Zr-huJ591, ^89^Zr-trastuzumab and ^89^Zr-pertuzumab IF, noise level of 5%, *rK*_*i*_ of 5·10^–3^ h^−1^ and *rV*_*T*_ of 0.2 are shown in Fig. 3, and results are listed in Table 2. Including a time point at 24 h p.i. reduced bias and variability in *V*_*T*_ for all three IF. Bias in *K*_*i*_ was reduced for ^89^Zr-huJ591 and remained similar for ^89^Zr-trastuzumab and ^89^Zr-pertuzumab. Variability in *K*_*i*_ remained similar for ^89^Zr-huJ591 and reduced slightly for ^89^Zr-trastuzumab and ^89^Zr-pertuzumab. Therefore, time point 24 h p.i. was included in all subsequent simulations.

**Table 2:** Smallest and largest percentage bias and variability of simulations with and without time point 24 h p.i. for both *K*_*i*_ and *V*_*T*_

	With 24 h		Without 24 h		With 24 h		Without 24 h	
	*K*_*i*_				*V*_*T*_			
	Smallest	Largest	Smallest	Largest	Smallest	Largest	Smallest	Largest
*Bias (%)*								
^89^Zr-huJ591	− 6.2	− 16.2	− 12.5	− 24.9	− 7.0	7.0	− 9.3	− 46.7
^89^Zr-trastuzumab	− 2.7	− 10.0	− 2.9	− 8.1	− 2.2	3.2	− 11.2	− 55.4
^89^Zr-pertuzumab	− 1.5	− 5.0	− 2.0	− 6.4	− 3.0	0.8	− 8.4	− 36.0
*Variability (%)*								
^89^Zr-huJ591	9.2	11.1	9.9	10.9	16.3	57.4	43.6	127.6
^89^Zr-trastuzumab	7.2	10.7	9.6	16.4	14.6	22.2	32.3	92.8
^89^Zr-pertuzumab	7.4	10.5	9.1	13.9	13.2	20.5	28.3	71.6

Simulations with ^89^Zr-pertuzumab as IF and 5% noise level showed that bias in *K*_*i*_ ranged from − 0.5% (absolute bias of − 5·10^–6^ for *K*_*i*_ = 1·10^−3^ and *V*_*t*_ = 0.1) to − 6% (absolute bias of − 1.1·10^−3^ for *K*_*i*_ = 20·10^−3^ and *V*_*t*_ = 0.5) and bias in *V*_*T*_ ranged from 2% (absolute bias of 0.01 for *V*_*t*_ = 0.5 and *K*_*i*_ = 1·10^−3^) to − 16% (absolute bias of − 0.016 for *V*_*t*_ = 0.1 and *K*_*i*_ = 1·10^−3^). Increasing the values for *rK*_*i*_ and *rV*_*T*_ resulted in increased variability in *K*_*i*_ and *V*_*T*_. Higher values for *rK*_*i*_ also increased bias in *K*_*i*_. However, bias in *K*_*i*_ resulting from increased *rV*_*T*_ and bias in *V*_*T*_ resulting from increased *rK*_*i*_ and *rV*_*T*_ remained similar, see Fig. 4.

Simulations with ^89^Zr-huJ591, ^89^Zr-trastuzumab and ^89^Zr-pertuzumab IF, *rK*_*i*_ of 1·10^−3^ h^−1^ and *rV*_*T*_ of 0.2 showed a threefold increase in variability in *K*_*i*_ and *V*_*T*_ with higher noise levels, bias remained similar. For ^89^Zr-huJ591, increasing the noise level from 5 to 15% increased variability in *K*_*i*_ (SE from 23.0 to 68.0% and from 30.0 to 90.6%, respectively) and variability in *V*_*T*_ (SE from 10.1 to 29.6% and 29.2 to 86.1%, respectively), while biases remained similar for *K*_*i*_ (from − 4.9 to − 5.1 and − 16 to − 16%, respectively) and *V*_*T*_ (from − 1.6 to − 2.3% and 2.3 to 1.8%, respectively). Results of the other two IFs showed the same pattern. The noise level dependency was similar for higher *rK*_*i*_ and *rV*_*T*_, however with higher bias and variability because of increased *rK*_*i*_ and *rV*_*T*_.

A decrease in AUC_*p*_ of the IF (in the order ^89^Zr-pertuzumab, ^89^Zr-trastuzumab, ^89^Zr-huJ591) resulted in increased bias in *K*_*i*_ and increased variability in *V*_*T*_ with increased *rK*_*i*_, see Fig. 5. For *rK*_*i*_ values of 20·10^−3^ h^−1^, bias in *K*_*i*_ also depended on the included time points, where the combinations 24, 48 and 192 h p.i. and 24, 144 and 192 h p.i. showed a larger underestimation of *K*_*i*_ of − 16% (absolute bias of − 3.2·10^−3^ for *K*_*i*_ = 20·10^−3^ and *V*_*t*_ = 0.1) for ^89^Zr-huJ591 IF as compared to − 10% for ^89^Zr-trastuzumab (absolute bias of − 2.0·10^−3^ for *K*_*i*_ = 20·10^−3^ and *V*_*t*_ = 0.1) and − 5.4% for ^89^Zr-pertuzumab IF (absolute bias of − 1.1·10^−3^ for *K*_*i*_ = 20·10^−3^ and *V*_*t*_  = 0.1). Decreased AUC_*p*_ of the IF also showed increased variability in *K*_*i*_ and *V*_*T*_ for increased *rV*_*T*_; however, bias remained similar.

Overall, when including time point 24 h p.i., there were only small differences found in bias and variability between different time point combinations. Only for high *K*_*i*_ values and the ^89^Zr-huJ591 IF (with faster clearance of the ^89^Zr-mAb from blood), bias in *K*_*i*_ and *V*_*T*_ showed a larger dependence on included time points, see Fig. 5. For all IFs, *rK*_*i*_, *rV*_*T*_, and time point combinations with noise level of 5%, percentage bias in *K*_*i*_ ranged from − 0.5 to − 16%.

## Discussion

This study evaluated the effect of the choice of imaging time points on the accuracy and precision of Patlak linearization for ^89^Zr-immuno-PET, considering different conditions. Simulations showed that inclusion of a PET scan and blood sample at 24 h p.i. improves accuracy and precision of Patlak results. Different combinations of later time points did not change the accuracy and precision in most cases. Moreover, increase in *rK*_*i*_, *rV*_*T*_ and noise level decreased accuracy and precision of Patlak results. Additionally, IFs with smaller AUC_*p*_ showed decreased accuracy and precision of Patlak results as compared to IFs with larger AUC_*p*_.

### *Underestimation of K*_*i*_

Bias in *K*_*i*_ was negative in all simulations. This can be explained by the shape of the IF in combination with the calculation of AUC_*p*_ in the Patlak equation [6]. In case the IF is fully described, for instance with a bi-exponential equation, determining the AUC_*p*_ by integration will result in the true value for AUC_*p*_. However, when only a finite set of points is known from the IF, determining the AUC_*p*_ will be based on trapezoidal numerical integration. For the simulations in this study, the latter applies, because data sampling is always finite. Since the activity concentration in plasma decreases over time in an exponential manner, the shape of the IF is curved downwards, leading to an overestimation of the AUC_*p*_ with trapezoidal numerical integration. The overestimated AUC_*p*_ increases the x-coordinates of the Patlak plot, which is AUC_*p*_/AC_*p*_, while the y-coordinates remain the same, because the ratio AC_*t*_/AC_*p*_ does not change. This results in a decreased positive slope of the Patlak plot, e.g., negative bias of *K*_*i*_.

### 24 h time point

Inclusion of time point 24 h p.i. showed to improve accuracy and precision of Patlak linearization. This is also due to the better assessment of the shape of the IF and the calculation of AUC_*p*_ as detailed before. The better the curve of the IF is described, by adding a time point in the most curved part of the IF, the more accurate the determination of AUC_*p*_ and Patlak parameters. One assumption for Patlak linearization is that equilibrium is reached between the ^89^Zr-mAb concentration in plasma and in the reversible tissue compartment, meaning that all fluxes are constant with respect to time [6]. In this study, activity concentrations in tissue were simulated by means of Patlak linearization and therefore were directly in equilibrium with activity concentrations in plasma. However, mAbs are relatively large proteins, therefore distribution inside the body takes relatively long, so tissue is not in rapid equilibrium with plasma [7]. Therapeutic antibodies cetuximab and trastuzumab showed approximately homogeneous distributions after 24 h p.i. in tumor-bearing mice [19]. For this reason, a period of 24 h was estimated to reach equilibrium between tissue and plasma. Additionally, from a practical point of view, it would not be possible to include time points after approximately 12 h, because PET scans should then be obtained outside working hours. Hence, time points before 24 h p.i. were not included in the simulations. This moment of equilibrium may differ between ^89^Zr-mAbs, and inclusion of a slightly earlier or later time point may be better depending on the mAb pharmacokinetics.

### Time point combinations

After inclusion of the 24 h p.i. time point, different time point combinations barely influenced Patlak results, which is advantageous from a practical perspective. Postponing a late imaging time point to a different day would not influence Patlak results. This is in contrast with obtaining the SUV, for which differences in the uptake time between injection and PET scan does influence the result, because SUV changes as a function of time [20]. In case the assumption of equal clearance between patients is true, comparisons of SUVs between patients would only be possible for PET scans that are obtained at the same uptake time post-injection [4]. Therefore, postponing a PET scan, resulting in different scan days for patients accompanied by different plasma activity concentrations, will influence SUV results. Apart from the ability to distinguish between reversible and irreversible, and potentially between non-specific and specific uptake of ^89^Zr-mAbs [8], the option to postpone a PET scan is another advantage of using Patlak linearization over using SUV in the quantification of ^89^Zr-immuno-PET.

### *Reference K*_*i*_* and V*_*T*_

Simulations showed that increasing *rK*_*i*_ and *rV*_*T*_ resulted in similar or increased bias and variability in both *K*_*i*_ and *V*_*T*_. As Patlak linearization is only applied when the assumption of irreversible uptake is met, *K*_*i*_ is never zero. Additionally, Jauw et al. [8] showed that organs without target expression have *K*_*i*_ values higher than zero, representing the catabolic rate of ^89^Zr-mAbs in healthy tissue. Values for *K*_*i*_ in this study are therefore all above zero.

### Noise levels

In this study, noise was approximated based on counting statistics, which resulted in noise increasing over time. This was similar to results from a study about noise-induced variability in PET imaging for ^89^Zr-immuno-PET, where recovery coefficients (RC) also increased over time from day 0 to day 6 [21]. RC was defined as 1.96*SD(%). RCs found for Kidney, lung, spleen and liver combined ranged from 2 to 11 [21], resulting in a maximum SD of approximately 5%. Similarly, SD derived from the RCs of tumor SUVpeak results in 15%. Simulations including TAC*s* with a 5% noise level may therefore represent biodistribution and TAC*s* with a 15% noise level may represent tumor uptake. Increasing the noise level from 5 to 15% only increased the variability, biases remained the same. Additionally, results of simulations with a noise level of 15% showed the same pattern as simulations with a 5% noise level and were chosen not to be presented.

### Input functions

The literature search provided five different ^89^Zr-mAb plasma IFs in patients, of which three were used for the simulations, while there are currently 119 therapeutic antibodies approved by the FDA [22]. However, these three ^89^Zr-mAb plasma IFs used in this study provide a wide range of clearances, covering substantial variability in IFs.

Simulations showed a dependency of Patlak results on the IF. For high *rK*_*i*_, accuracy and precision in Patlak results decreased with AUC of the IF (i.e., faster clearance), in the following order: ^89^Zr-pertuzumab, ^89^Zr-trastuzumab and ^89^Zr-huJ591. A decrease in AUC_*p*_ will result in lower x-coordinates of the Patlak plot, thereby bringing the datapoints closer together resulting in higher contribution of noise. The AUC_*p*_ is the integral of the activity concentration in plasma, which is the total ^89^Zr-mAbs present in the plasma cumulated over time from injection to moment of PET scan. For the simulations, the IF and *rK*_*i*_ were regarded as two independent variables; however, they are physiologically related. For IFs with lower AUC_*p*_, so faster clearance, higher irreversible uptake in tissue (*rK*_*i*_) is expected. However, simulations showed that a higher *rK*_*i*_ for the ^89^Zr-huJ591 IF resulted in decreased accuracy of *K*_*i*_ (− 16%) and precision of *V*_*T*_ as compared to the other IFs. This indicates that accuracy and precision of Patlak results are worse for ^89^Zr-mAbs with faster clearance combined with higher irreversible uptake. However, for volumes of interest showing high irreversible uptake, a bias in *K*_*i*_ of − 16% would not change the (clinical) decision-making based on the data, because the observed irreversible uptake would still be high.

This study considers input functions with binding of targets on cells that do not redistribute during the course of the PET studies (HER2 for trastuzumab and pertuzumab, and PSMA for huJ591). However, the usefulness of Patlak linearization may be limited in case of ^89^Zr-mAbs that bind to mobile immune cells, such as the PD-1 receptors on T-cells. In order to apply Patlak linearization, an equilibrium between reversible processes is assumed as well as a constant density of specific targets or receptors. Changes in receptor availability during the course of the study may introduce inaccuracies in Patlak linearization. Yet, Patlak analysis also has several advantages over SUV. Patlak linearization can also be applied with higher mass dose. However, there are two phenomena that need to be considered. First of all, higher mass doses will result in slower plasma clearance. Patlak linearization takes into account the mAb concentration in plasma (or input function) and no assumptions are required with regard to (changes in) plasma clearance as the measured plasma kinetics are used. Secondly, a higher administered mass dose will result in lower uptake in tissue of interest. Patlak linearization is still valid with higher mass doses; however, lower K_i_ values are expected because of the reduced receptor availability/higher receptor or target occupancy. Also, Menke-van der Houven van Oordt et al. [9] showed in their study that Patlak linearization applied to PET imaging data with different administered mass doses allows evaluation of the optimal therapeutic dose. By plotting the Patlak K_i_ values against increasing mass doses a S-curve can be obtained. K_i_ values decrease because of target binding competition between labeled and unlabeled mAbs. This curve allows evaluation of the 50% inhibitory mass dose (ID50). The ID50, the dose at which 50% of the targets are occupied, can be used in establishing the optimal therapeutic dose [9].

## Conclusion

This study evaluated the effect of imaging time points on the accuracy and precision of Patlak results, for different IFs, imaging time points, noise levels, and tissues with different levels of reversible and irreversible uptake. Quantification of ^89^Zr-immuno-PET using Patlak linearization can generate accurate results within − 0.5% and − 16% bias for *K*_*i*_ (at a 5% noise level), provided that a 24 h p.i. time point and two later time points are included. The exact timing of the two other scans and samples is, however, not critical as opposed to SUV-based quantification.

## Supplementary Information

**Additional file 1.** In-house written MATLAB code for Patlak linearization. The in-house written MATLAB function provided in Supplemental 1 was used for Patlak linearization calculations.

## Data Availability

All data and scripts generated during the current study are available from the corresponding author on reasonable request.
