# Supplementary material for: Optimal imaging time points considering accuracy and precision of Patlak linearization for 89Zr-immuno-PET: a simulation study
Source: EJNMMI Res. 2022 Sep 5;12:54. doi: 10.1186/s13550-022-00927-6 (PMC9445120; doi:10.1186/s13550-022-00927-6)
Supplement: Supplementary file 1 — Additional file 1. In-house written MATLAB code for Patlak linearization. The in-house written MATLAB function provided in Supplemental 1 was used for Patlak linearization calculations. [file 13550_2022_927_MOESM1_ESM.pdf]

Optimal imaging time points considering accuracy and precision of Patlak linearization for ^{89}Zr-immuno-PET: a simulation study

Jessica E. Wijngaarden[1], Marc C. Huisman[1], Johanna E. E. Pouw[2], C. Willemien Menke-van der Houven van Oordt[2], Yvonne W.S. Jauw[1,3], Ronald Boellaard[1]

Author's affiliations

[1] Amsterdam UMC, Vrije Universiteit Amsterdam, Department of Radiology and Nuclear Medicine, Cancer Center Amsterdam, Amsterdam, The Netherlands

[2] Amsterdam UMC, Vrije Universiteit Amsterdam, Department of Medical Oncology, Cancer Center Amsterdam, Amsterdam, The Netherlands

[3] Amsterdam UMC, Vrije Universiteit Amsterdam, Department of Hematology, Cancer Center Amsterdam, Amsterdam, The Netherlands

First and corresponding author:

Name: Jessica E. Wijngaarden

E-mail: j.e.wijngaarden@amsterdamumc.nlAddress: De Boelelaan 1117, 1081 HV Amsterdam, The Netherlands

Telephone number: 020-4442863

Fax number: 020-4444329

SUPPLEMENTAL 1

The in-house written MATLAB function below was used for the Patlak linearization calculations. Activity concentration in plasma (`ACp`), activity concentration in tissue (`ACt`) and time between moment of injection and moment of measurement (`time`) were used as input. The function provides as output the linear regression fit (`lin_fit`) obtained from the build-in MATLAB function polyval, the calculated Patlak simulated K_i value (`Ki`), the calculated Patlak V_T value (`Vt`) and the R-squared of the linear regression fit (`Rsqr`).

```
function [lin_fit,Ki,Vt,Rsqr] = patlak_calc(ACp,ACt,time)
%% Patlak linearization: ACt/ACp = Ki*(AUCp/ACp)+Vt

% AUCp: area under the ACp curve from time of injection to time of
measurement
AUCp = cumtrapz(time,ACp); % numerical integration

% Calculate Patlak x and y values
ACtACp = ACt./ACp;
AUCpACp = AUCp./ACp;

% Calculate Ki and Vt with fitting linear regression (based on least
squares)
stFit = find(time>=24); %indices of time more than 24 hours
stFit = stFit(1);
[coeff,S] = polyfit(AUCpACp(stFit:end),ACtACp(stFit:end),1); %first order
because linear, ignore first datapoint
[lin_fit] = polyval(coeff(:),AUCpACp(stFit:end),S);

Ki = coeff(1)*1000; %from mL/g/h to microL/g/h
Vt = coeff(2);

% R^2 of linear fit
Rsqr = 1-(S.normr/norm(ACtACp-mean(ACtACp)))^2;
end
```
